# Last general practitioner consultation during the final month of life: a national medical record review of suicides in Sweden

**DOI:** 10.1186/s12875-024-02498-y

**Published:** 2024-07-15

**Authors:** Nina Palmqvist Öberg, Sara Probert Lindström, Erik Bergqvist, Anna Ehnvall, Tabita Sellin, Anne Stefenson, Charlotta Sunnqvist, Margda Waern, Åsa Westrin

**Affiliations:** 1 Department of Clinical Sciences, Psychiatry, Lund University, Lund, SE-221 84 Sweden; 2 Office of Psychiatry and Habilitation, Region Skåne, Lund, SE-221 85 Sweden; 3 Psychiatric In-patient Clinic, Hallands Sjukhus Varberg, Region Halland, Varberg, SE-432 81 Sweden; 4 Department of Psychiatry and Neurochemistry, Institute of Neuroscience and Physiology, University of Gothenburg, Gothenburg, SE-413 45 Sweden; 5 Psychiatric Out-patient Clinic, Region Halland, Varberg, SE-432 43 Sweden; 6 Faculty of Medicine and Health, University Health Care Research Center, Örebro University, Örebro, SE-701 82 Sweden; 7 National Centre for Suicide Research and Prevention of Mental Ill-Health (NASP), Karolinska Institute, Stockholm, SE-17177 Sweden; 8 The Region Skåne Committee on Psychiatriy, Habilitation and Technical Aids, Lund, Sweden; 9 Psychosis Clinic, Region Västra Götaland, Sahlgrenska University Hospital, Gothenburg, 41345 Sweden

**Keywords:** Suicide, Last contact, Primary care contact

## Abstract

**Objectives:**

Individuals who die by suicide often consult their general practitioners (GPs) in their final weeks of life. The aim of this study was to gain a deeper knowledge of the clinical characteristics and GP assessments and treatments among individuals who consulted their GPs during the month preceding suicide. Further, we compared these features in those with and without contact with psychiatric services (PC and NPC, respectively) during the two years that preceded the suicide.

**Design:**

A nationwide retrospective explorative study investigating medical records.

**Setting:**

Primary care in Sweden.

**Participants:**

Individuals who died by suicide in Sweden in 2015 with a GP visit within 30 days of death.

**Results:**

The study cohort corresponds to one fifth (*n* = 238) of all suicides that occurred in Sweden in 2015 (*n* = 1179), representing all those with available primary care records showing contact with a GP during the final 30 days of life (NPC: *n* = 125; PC: *n* = 113). The mean age was 58 years ± 19. Patients in the NPC group were older (NPC: 63 years ± 19 vs. PC: 53 years ± 18, *p* < 0.0001) and presented psychiatric symptoms less often (NPC: 50% vs. PC: 67%, *p* < 0.006). Somatic symptoms were as common as psychiatric symptoms for the whole sample, being present in more than half of individuals. Suicide risk was noted in only 6% of all individuals. Referral to psychiatric services occurred in 14%, less commonly for the NPC group (NPC: 6% vs. PC: 22%, *p* < 0.001). Cardiovascular or respiratory symptoms were noted in 19%, more often in the NPC group (NPC: 30% vs. PC: 6%, *p* < 0.001), as were diagnoses involving the circulatory system (all 10%, NPC:14% vs. PC: 5%, *p* < 0.020).

**Conclusion:**

A high level of somatic symptoms was observed in primary care patients both with and without psychiatric contact, and this might have influenced GPs’ management decisions. Psychiatric symptoms were noted in two thirds of those with psychiatric contact but only half of those without. While GPs noted psychiatric symptoms in over half of all individuals included in the study, they seldom noted suicide risk. These findings highlight the need for increased attention to psychiatric symptoms and suicide risk assessment, particularly among middle-aged and older individuals presenting with somatic symptoms.

**Strengths and limitations of this study:**

The National Cause of Death Register has excellent coverage of suicide deaths and access to medical records was very good. The medical record review provided detailed information regarding primary care utilization before death by suicide. Because of the lack of statistical power, due to the limited number of persons with GP contact during the last month of life, we chose not to correct for multiple comparisons. Our study approach did not capture the reasons behind GPs’ documentation of elevated suicide risk. No systematic inter-rater reliability (IRR) testing was made, however, reviewers received training and continuous support from the research group.

**Supplementary Information:**

The online version contains supplementary material available at 10.1186/s12875-024-02498-y.

## Introduction

International studies of Western societies show that a large proportion of individuals who die by suicide make contact with health care in the year before suicide, and many make contact within the final weeks [1, 2]. Most of these contacts occur outside of psychiatric specialist care in medical specialist care or primary health care [3]. As previously shown by our research group, this is also the case for Sweden, a high-income country with publicly funded health care. Here about 60% of people who die by suicide have one or more health care contacts in the last 4 weeks of life, with the older adults having contact with primary care to a greater extent than younger [4]. This aligns with a recent study from Wales reporting that primary health care is the final point of medical contact for a large majority of individuals who die by suicide [5]. Furthermore, a case-control study from Ireland recently found that individuals who died by suicide were more likely than controls to have high rates of GP consultation [6]. Thus, primary care is a key venue for suicide prevention, after psychiatric symptoms and suicidal ideation have been identified, as reflected in a large review of suicide prevention by O’Connor et al. [7]. Nevertheless, identifying these individuals can be challenging, particularly given the large number of patients in primary care settings, where limited appointment times may hinder the detection of mental health issues. Furthermore, the reasons for primary care visits are diverse, and mental health concerns may be overshadowed by somatic complaints [8]. Previous international studies have reported that between 30% [9] and 62% [10] of individuals who die by suicide present psychiatric symptoms at their final primary care consultation. The proportion presenting somatic symptoms at the last visit differs among studies, with figures ranging from 22% [10] to 44% [11], with the latter figure based not only on primary health care but also on other medical settings. In a Taiwanese study, only 20% of individuals who visited a non-psychiatric health service in the month preceding suicide received a psychiatric diagnosis [12].

According to a British study [13], individuals who made contact only with primary health care in the year prior to suicide were more likely to be working, in a relationship, have no known psychiatric disorder and have a lower assessed suicide risk, compared to individuals that were referred to specialized psychiatric care. However, it is difficult to draw clinical conclusions from this study since it did not describe psychiatric or somatic symptoms nor provide data on clinical actions and treatments, other than referrals to specialist psychiatric care.

In summary, we have little knowledge, both internationally and in the Swedish context, about how individuals who will die by suicide within a short period of time after having met with a GP are perceived by the GP, and what treatment plans are made for them in relation to this. Equally unknown is whether individuals with prior psychiatric contacts are perceived differently at this visit and how this affects the planning of their care. Such information could inform the development of more effective strategies for identifying at-risk individuals within primary care settings and enhancing group-level preventive interventions.

## Aims

The aim of this study was to explore the clinical characteristics such as symptoms, assessments and treatments of individuals who die by suicide shortly after a primary care consultation in Sweden. Also, we wanted to compare the sociodemographic characteristics, clinical features and planned treatments of primary care patients with and without previous contact with psychiatric services. Understanding these differences might also help GPs in identifying psychiatric problems in their patients.

## Methods

### Design

This study is part of the nationwide research project “Retrospective investigation of health care utilization of individuals who died by suicide in Sweden in 2015”, where we examined medical records from the two years preceding suicide, including psychiatric care, primary care and somatic care, from all major healthcare providers in the public and private sectors, as well as from non-major health care providers (e.g. private GPs offices). All the data was gathered in a review template based on the Swedish Psychiatric Association’s guidelines for the assessment and treatment of suicidal patients [14]. The nationwide research project is further described elsewhere [4]. In the present study, clinical data was drawn exclusively from the last GP visit prior to suicide and included, for example, symptoms, diagnoses, assessments and planned treatment as described in the variable list.

### Study population/ setting

The overall project is based on the data of individuals (*n* = 1179) who died by confirmed suicide (as listed in the Swedish Cause of Death Register) in Sweden in 2015. The total population of Sweden on December 31, 2015 was 9,851,017 [15]. The current study focuses on those with a record of GP contact within 30 days of death (*n* = 238).

### Data collection

Personal identification numbers and variables from the Cause of Death register were requested from the Swedish National Board of Health and Welfare for all suicide decedents (ICD-10 codes X60–X84, intentional self-harm) [16] from January 1 to December 31, 2015. The Swedish Cause of Death Register is a high-quality source of data suitable for research purposes [17]. At the time of data request, 1179 suicides were listed in the register. Cases added later in connection with an update of the register (*n* = 7) were not included in the current study. Confidentiality agreements were made with each region for the access to the medical records of the individuals. The medical records were reviewed by mental health professionals who were trained in data extraction by the research group to ensure reliability. All data extractors had access to support from the research group throughout the process. Only the principal investigator of the main study, the local representative responsible for health care in each of Sweden’s 21 regions, the data extractors and the research nurse at the research unit had access to the personal data and personal identification numbers of participants. Each deceased person was assigned a code, which was written on the templates used for collecting data from medical records. Separate templates were used for primary care, specialist psychiatric care, and each relevant somatic specialist care unit. The data files from the different regions were merged into a master file by the project statistician. Personal identification number, death date and region of residence were extracted from the Cause of Death Register. All other data were collected during the medical record review.

**Fig. 1:**
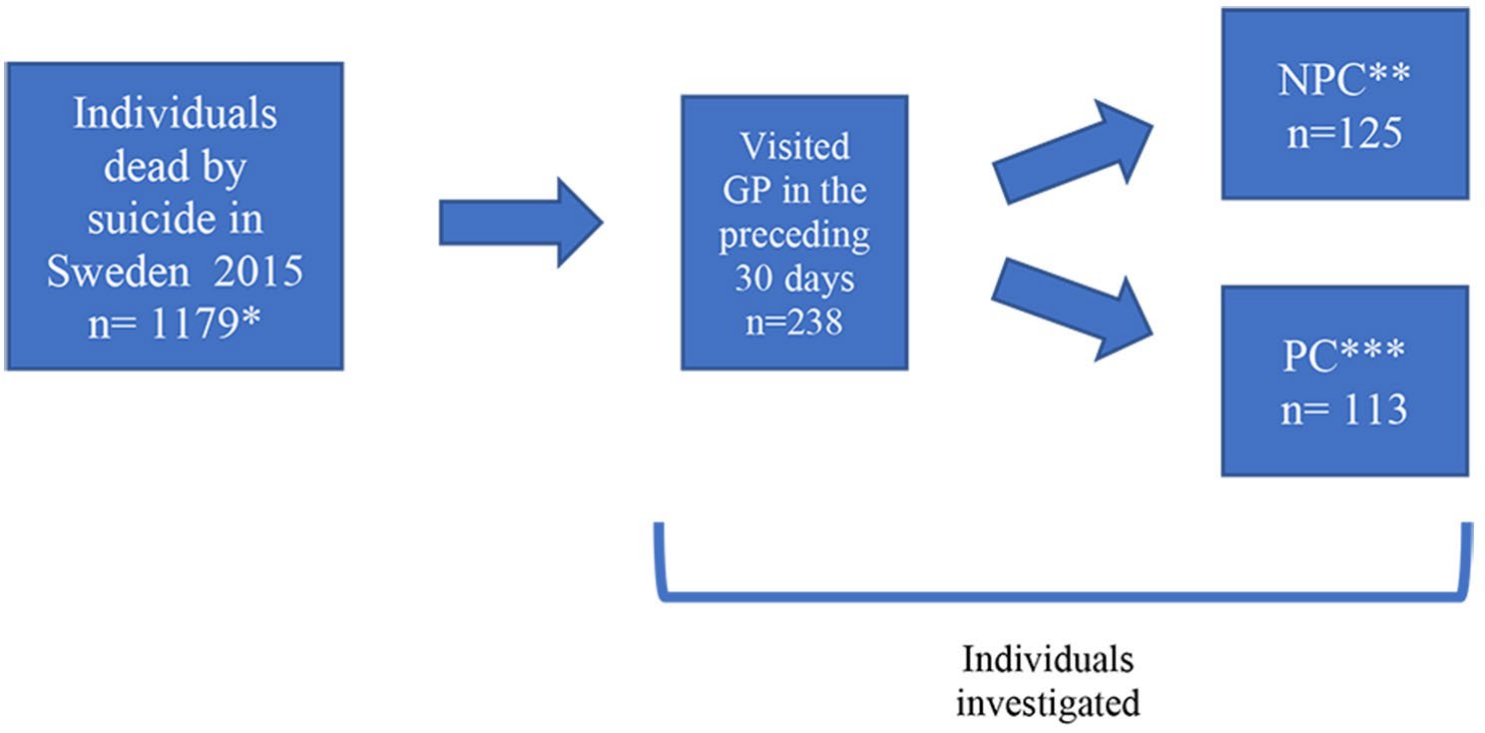
Flowchart of individuals selected in the study. * At the time of data request, 1179 suicides were recorded in the Swedish Cause of Death Register. ** The NPC (No Psychiatric Contact) group consisted of individuals who did not have any contact with psychiatric services during the last two years of life (prior to the last GP contact). *** The PC (Psychiatric Contact) group consisted of individuals who did have contact with psychiatric services during their last two years of life (prior to their last final GP contact)

The record review showed that over half (125 of the 238) of the individuals who had visited a GP in the 30 days prior to suicide did not have contact with psychiatric services in the previous two years. For the purposes of this paper, this group was called the No Psychiatric Contact (NPC) group. This included 10 persons with first psychiatric contact *after* the final GP visit. All others (*n* = 113) were included in the Psychiatric Contact (PC) group (Fig. 1). To capture individuals who were more likely to be further along in the suicide process we focused on primary health care contact within the 30 days preceding death. In terms of contact with psychiatric services, the selection of individuals with contact within the previous two years was to capture those who had been relatively recently in contact with psychiatric services. For the purposes of this article, the term “contact with psychiatric services” means visiting any profession employed in psychiatric specialist care. The individuals came from all of Sweden’s 21 regions, with no region excluded.

### Selected variables

In this study, the selected variables of interest were as follows:

- gender (male or female).
- occupation (any full-time or part-time work, studies, or participating in an employment agency project or similar, as noted in the medical records during the previous two years, as close to death as possible).
- civil status (as noted in the medical records during the previous two years, as close to death as possible).
- psychiatric symptoms and signs at the last GP consultation.
- somatic symptoms and signs at the last GP consultation.
- suicide risk (whether or not the patient was reported as having an elevated suicidal risk by the GP in the medical record with no further information on how the GP arrived at this conclusion).
- diagnoses (ICD-10 codes) as assigned by GP at last consultation (multiple diagnoses were allowed).
- investigation, referrals, planned follow-up and treatments at the last GP consultation.
- prescribed medication as listed in the primary care record at the time of death, coded by Anatomical Therapeutic Chemical (ATC) number.

Psychiatric symptoms and signs as well as somatic symptoms were predefined categories in the templates. For psychiatric symptoms, the category “signs of depressiveness” encompassed the individual presenting with sadness or a depressive mood; this category was constructed to capture if the GP had noticed signs or symptoms of depression. The category of “psychiatric investigation in primary care” comprised, for example, an investigation by a social counsellor or psychologist, contact between GP and relatives for diagnostic purposes or to better understand the person’s condition, or using diagnostic rating scales. A “somatic investigation” might be making specific physical examinations or taking laboratory tests.

The variables are further described in Tables 1, 2, 3, 4 and 5.

### Statistical analyses

For categorical variables (i.e. background characteristics, somatic and psychiatric symptoms, suicide risk, diagnoses and planned treatment), between-group analyses were conducted using frequency description and χ^2^ tests. The continuous variable (age) was tested with the independent samples t-test. The results were considered statistically significant at a two-sided *p* < 0.05. SPSS Statistics 28 was used for all analyses. Given limited power with the small number of individuals in the study, we chose not to correct for multiple comparisons.

### Patient and public involvement

None.

## Results

### Characteristics of the study population

The characteristics of the study population are shown in Table 1.

**Table 1:** Characteristics of the study population (*n* = 238): individuals who died by suicide in Sweden in 2015 who visited a general practitioner in the 30 days preceding suicide

	All individuals (*n* = 238)	No contact with psychiatric services in the previous two years (NPC) (*n* = 125)	Contact with psychiatric services in previous two years (PC) (*n* = 113)	χ^2^	p*
Age, years at the time of suicide (Mean ± SD)	58.1 ± 19	63.3 ± 18.7	52.5 ± 17.6	4.574	< 0.001
Gender (n) %					
Male	162 (68.1)	92 (73.6)	70 (61.9)	3.708	0.054
Female **	76 (31.9)	33 (26.4)	43 (38.1)		
Occupation***					
(n) %					
Yes	81 (39.7)	35 (32.4)	46 (47.9)	15.886	< 0.001
No (Unemployed)	30 (14.7)	10 (9.3)	20 (20.8)		
Retired (age)	93 (45.6)	63 (58.3)	30 (31.3)		
Civil status****					
% (n)					
Married/partner	81 (39.9)	49 (46.2)	32 (33.0)	4.085	0.090
Divorced/no partner	101 (49.8)	45 (42.5)	56 (57.7)		
Widow/widower	21 (10.3)	12 (11.3)	9 (9.3)		

*Differences between the NPC and PC groups were tested with independent samples t-tests (age) and chi-square analysis (all other variables), df = 1(gender), df = 2 (occupation, civil status)

**including individuals who had completed gender transition

***occupation was operationalized as working, studying, or taking part in a work rehabilitation project or other project through the Swedish Employment Agency, *n* = 204, missing data in 14% due to unavailable information. Retired (age) stands for a person being retired because of age and not for example because of disability

****n = 203, missing data in 15% due to unavailable information

One fifth of all individuals who died by suicide in Sweden had a GP consultation during the final month of life. The mean age of these was 58 years, with the NPC group significantly older than the PC group. Accordingly, a higher proportion of retired people was found in the NPC group than in the younger PC group. There were more men than women across the whole sample. The proportion of women was numerically higher in the PC group but the difference did not reach significance. 39% of the whole sample had an occupation (work, studies or participating in an employment agency project or similar) listed in their medical records. The PC group had a higher proportion of unemployed individuals. 40% lived with a partner, with no statistical difference between the groups.

### Symptoms and signs

Psychiatric symptoms were noted in more than half of all, and in about half of the individuals in the NPC group and two-thirds of those in the PC group (Table 2). Depressive mood was the most common psychiatric symptom noted by the GP in both groups and was present in 42% of all individuals. Anxiety was significantly more common in the PC group than in the NPC group. There were no significant differences between the two groups in symptoms or signs of depressiveness, sleep disturbances, substance abuse, crisis, memory problems/confusion or other psychiatric symptoms. Suicide risk was considered elevated by the GP in 6% of all individuals (*n* = 14) (NPC: 3% vs. PC: 9%, *p* = 0.064, χ^2^ 3.422).

Table 2 shows that somatic symptoms or signs were reported by the GP in 61% of the individuals, with no statistical differences between the groups. Cardiovascular (e.g. chest pain or palpitations) or respiratory symptoms or hypertension were reported significantly more often in the NPC group. Physical pain was mentioned in the medical record in 32% of all patients but did not significantly differ between the two groups. There were no significant differences in fatigue or loss of energy, neurological signs or dizziness, weight loss or loss of appetite or other somatic symptoms or signs between the two groups.

**Table 2:** Symptoms and signs noted in the medical records at final consultation with a general practitioner in the 30 days preceding suicide (*n* = 238)

	All individuals (*n* = 238) *n* (%)	No contact with psychiatric services in the previous two years (NPC) (*n* = 125) *n* (%)	Contact with psychiatric services in the previous two years (PC) (*n* = 113) *n* (%)	χ^2^	p*
Psychiatric symptoms and signs	138 (58.0)	62 (49.6)	76 (67.3)	7.595	0.006
Depressive mood	98 (41.2)	45 (36.0)	53 (46.9)	2.913	0.088
Anxiety	93 (39.1)	39 (31.2)	54 (47.8)	6.859	0.009
Sleeping disturbances	69 (29.0)	38 (30.4)	31 (27.4)	0.254	0.614
Substance abuse	15 (6.3)	6 (4.8)	9 (8.0)	1.006	0.316
Crisis	26 (10.9)	9 (7.2)	17 (15.0)	3.753	0.053
Memory problems/ confusion	13 (5.5)	8 (6.4)	5 (4.4)	0.448	0.503
Elevated suicide risk**	14 (6)	4 (3)	10 (9)	3.422	0.064
Other psychiatric symptoms***	35 (14.7)	16 (12.8)	19 (16.8)	0.762	0.383
Somatic symptoms and signs	145 (60.9)	81 (64.8)	64 (56.6)	1.661	0.197
Cardiovascular/ Hypertonia/ Respiratory symptoms****	44 (18.5)	37 (29.6)	7 (6.2)	21.574	< 0.001
Physical pain Fatigue, energy loss	76 (31.9) 37 (15.5)	41 (32.8) 18 (14.4)	35 (31.0) 19 (16.8)	0.091 0.263	0.763 0.608
Neurological or dizziness	24 (10.1)	17 (13.6)	7 (6.2)	3.589	0.058
Loss of appetite/ weight loss	30 (12.6)	16 (12.8)	14 (12.4)	0.009	0.924
Other somatic*****	55 (23.1)	32 (25.6)	23 (20.4)	0.919	0.338

*Differences between the groups NPC and PC were tested with chi-square analysis, df = 1 for all

**Physician assessed

***Other psychiatric symptoms included symptoms of psychosis, suicidal thoughts and/or plans, and other symptoms

****Cardiovascular/ Hypertonia/ Respiratory symptoms: a cluster of symptoms, where the individual had at least one of the symptom groups. Cardiovascular symptoms could be for example palpitations, a feeling of pressure in the chest and respiratory symptoms for example coughing, feeling of breathlessness or other respiratory symptoms

*****Other somatic symptoms included gastrointestinal, urinary tract or infection symptoms

### Diagnoses noted at the last GP contact

Clinical diagnoses recorded at the last GP contact are given in Table 3. According to the medical records, 44% of individuals had a psychiatric diagnosis, with diagnoses in F30–F39 (mood disorders) and in F40–F48 (neurotic, stress-related and somatoform disorders) being the most common diagnoses. There were no significant differences in the proportions of these diagnoses in those with and without psychiatric contact.

Table 3 shows that most somatic diagnoses were equally distributed in both groups, with more than half (57%) of the individuals having at least one somatic diagnosis. Common diagnoses were musculoskeletal (M00-M99) and the descriptive “Symptoms and Signs” (R00–R99). Diagnoses involving the circulatory system (I00–I99) were significantly more common in the NPC group than in the PC group.

**Table 3:** Diagnosis at the final consultation with the general practitioner in the 30 days preceding suicide (*n* = 238)

	All individuals (*n* = 238) *n* (%)	No contact with psychiatric services in the previous two years (NPC) (*n* = 125) *n* (%)	Contact with psychiatric services in the previous two years (PC) (*n* = 113) *n* (%)	χ^2^	p*
Any psychiatric diagnosis**	105 (44.1)	49 (39.2)	56 (49.6)	2.582	0.108
F30–F39 Mood disorders	55 (23.1)	25 (20.0)	30 (26.5)	1.432	0.231
F40–F48 Neurotic, stress-related, somatoform disorders	54 (22.7)	25 (20.0)	29 (25.7)	1.085	0.298
Other psychiatric diagnoses***	26 (10.9)	9 (7.2)	17 (15.0)	3.753	0.053
Any somatic diagnosis****	135 (56.7)	77 (61.6)	58 (51.3)	2.551	0.110
I00–I99 Dis. of the circulatory system	24 (10.1)	18 (14.4)	6 (5.3)	5.409	0.020
M00–M99 Dis. of the musculoskeletal system and connective tissue	32 (13.4)	14 (11.2)	18 (15.9)	1.141	0.286
R00-R99 Symptoms signs*****	34 (14.3)	20 (16.0)	14 (12.4)	0.632	0.427
Other somatic diagnoses******	87 (36.6)	48 (38.4)	39 (34.5)	0.387	0.534

*Differences between the NPC and PC groups were tested with chi-square analysis, df = 1 for all

** Diagnoses from Chap. 5 of the ICD-10, ‘Mental and behavioural disorders’

***Other psychiatric diagnoses; F00–F09 Organic disorders, F10–F11 Mental and behavioural disorders due to psychoactive substance abuse, F20–29 Schizophrenia, schizotypal and delusional disorders., F50–F59 Behavioural syndromes, F60–F69 Personality disorders, F70–F98 Developmental disorders such as intellectual disability, autism, and attention deficit hyperactivity disorder (ADHD)

****Diagnoses outside of Chap. 5 of the ICD-10

***** Symptoms, signs and abnormal clinical findings, not elsewhere classified

******Other somatic: A00–B99 Infectious diseases, C00–D48 Neoplasms, D50–D89 Diseases of blood and blood-forming organs, E00–E90 Endocrinological, G00–G99 Nervous system, H00–H59 Diseases of Eye, H60–H95 Ear, J00–J99 Respiratory system, K00– K93 Digestive system, L00–L99 Skin, N00–N99 Genitourinary system, O00–O99 Pregnancy, birth and puerperium, P00–P96 Perinatal, Q00–Q99 Congenital, S00–T98 Injury, poisoning etc., V01–Y98 External causes of morbidity and factors influencing health and contact with health care

### Investigations, referrals, and treatments at the last GP visit

An overview of the investigations, referrals and treatments at the last GP visit is given in Table 4. Half of all individuals had somatic investigations performed or planned at primary care and this was significantly more common in the NPC group than in the PC group. However, there was no significant difference in referral to specialist somatic care between the two groups. A psychiatric investigation in primary care was noted in about 8% of the consultations, with no statistical difference between the groups, but referral to psychiatric services was made for a larger proportion of the PC group than of the NPC group. 37% of all individuals were given or planned to get treatment for their mental health problems at the final visit to their GP.

**Table 4:** Overview of investigation, referrals and treatment at last consultation with the general practitioner in the 30 days preceding suicide (*n* = 238)

	All individuals (*n* = 238) *n* (%)	No contact with psychiatric services in the previous two years (NPC) (*n* = 125) *n* (%)	Contact with psychiatric services in the previous two years (PC) (*n* = 113) *n* (%)	χ^2^	p*
Somatic investigation in primary care	123 (51.7)	75 (60.0)	48 (42.3)	7.297	0.007
Referral to secondary somatic care	49 (20.6)	28 (22.4)	21 (18.6)	0.529	0.467
Psychiatric investigation primary care	20 (8.4)	10 (8.0)	10 (8.8)	0.056	0.814
Any mental health treatment in primary care**	88 (37.0)	48 (38.4)	40 (35.4)	0.229	0.632
Referral to secondary psychiatric care	33 (13.9)	8 (6.4)	25 (22.1)	12.286	< 0.001

*Differences between the NPC and PC groups were tested with chi-square analysis, df = 1 for all

** Mental health treatment in primary care was an independent variable in the research templates, consisting of for example medication, psychotherapy, follow-up visits within primary care etc

Almost half of the individuals were being treated with antidepressants at the time of suicide, according to their medication list in the primary health care records. However, there were no statistical differences in the prescription of antidepressants, anxiolytics/hypnotics or psychotherapy/counselling at the primary care level between the two groups (Table 5).

**Table 5:** Primary care follow-up and treatment of mental health problems at the last consultation with the general practitioner in the 30 days preceding suicide (*n* = 238)

	All individuals (*n* = 238) *n* (%)	No contact with psychiatric services in the previous two years (NPC) (*n* = 125) *n* (%)	Contact with psychiatric services in the previous two years (PC) (*n* = 113) *n* (%)	χ^2^	p*
Revisit planned within one week	29 (12.2)	15 (12.0)	14 (12.4)	0.008	0.927
Antidepressant medication**	109 (45.8)	55 (44.0)	54 (47.8)	0.343	0.558
Medication anxiolytic/hypnotic***	129 (54.2)	69 (55.5)	60 (53.1)	0.106	0.745
Psychotherapy/ supportive counselling	38 (16.0)	19 (15.2)	19 (16.8)	0.115	0.738

*Differences between the groups were tested with chi-square analysis, df = 1 for all

**N06A, ***N05B, N05 According to the individual’s medication list in their primary care record at the time of suicide. Medication for ADHD, substance abuse or neuroleptics was too rare to perform group analyses and no medication for dementia was found across the whole sample

A descriptive analysis of the 33 individuals who were referred to psychiatric specialist care showed that 30 (91%) had psychiatric symptoms noted by the GP, 27 (82%) a psychiatric diagnosis and 10 (30%) a notation of suicide risk as elevated. Their mean age was 52.4 years ± 14, and the gender proportion 22 males to 11 females.

Of the 14 individuals (11 males and 3 females) in the whole sample that had a notation of elevated suicide risk at their last GP consultation 10 were referred to specialist psychiatric care. Seven of them had a prescribed psychopharmacological treatment, 2 had ongoing or planned psychotherapy or counselling and 3 had planned follow-up in primary care within a week.

## Discussion

### Main findings

Among persons who died by suicide in Sweden, one-fifth had contact with a GP during their final month of life. Notably, less than half of these had psychiatric contact during the two years prior to the last GP consultation. There was an age difference; persons in the NPC group were older. Somatic symptoms were as common as psychiatric in the cohort at the final GP consultation and were noted in more than half. Half of the individuals in the NPC group had documented psychiatric symptoms compared with two thirds of those in the PC group. Less than half of the total cohort received a psychiatric diagnosis. At the final consultation one third were prescribed treatment of mental health problems at the primary care level. One fifth were referred to psychiatric specialist services; this was more common among persons with a history of previous psychiatric contact.

### GP contacts

The proportion of individuals who had contact with their GP in our study (20%) was notably lower than was reported in a recent study from France [18] and in an international systematic review [19]. In these studies, around 45% had contact with primary care in the month that preceded suicide. Part of the discrepancy may be explained by the fact that our study did not include other forms of primary care contacts. Including interactions with other primary health care professionals such as nurses, psychologists, psychotherapists and counsellors would yield an estimated 31% [4]. This figure is still somewhat low in comparison to other studies, and this could potentially be understood by differences in health care availability and organization. Psychiatric care is relatively available in the Scandinavian countries, and reports from both Sweden and Norway show lower rates of GP contacts and higher rates of contact with psychiatric services prior to suicide compared to other nations [4, 20].

### Psychiatric symptoms and diagnoses

Within our study cohort, 44% of all individuals received a psychiatric diagnosis at the last GP consultation. This figure is similar to that reported by Ahmedani et al. in a large US study, in which approximately half of the individuals who died by suicide received a mental health diagnosis during the last year [21]. It should be noted that the latter study included various health care contexts, while ours applies to primary care only. Previous research applying the psychological autopsy approach has indicated that more than 90% of individuals who die by suicide have a psychiatric illness [19], suggesting a need for clinician training. In line with this a recent systematic review by Mann et al. describes evidence for suicide prevention by training GP: s in recognizing and treating depression [22]. Our study highlights the need for such interventions, as well as interventions for to identify and mitigate anxiety, as such symptoms were frequently present in our suicide cohort [23].

### Somatic symptoms and diagnoses

In our study, over 60% of all individuals presented with somatic symptoms, a similar proportion to that noted for psychiatric symptoms. The high proportions of somatic symptoms is in line with a previous report from Finland where almost half of the individuals reported somatic symptoms at the last visit before suicide [11]. In our study the NPC group underwent somatic investigations more frequently at their final GP consultation, which to our knowledge is a new finding. This emphasis on somatic symptoms in the NPC group may be attributed to their age and somatic comorbidity, which are established risk factors for suicide [24].

Notably, cardiovascular symptoms, hypertension and respiratory symptoms were more prevalent in the NPC group. Although age adjustment was not feasible due to the small sample size, the 10-year disparity in mean age between the groups likely accounts for some of the variance in the prevalence of these somatic symptoms. Similarly, a previous study on the diagnoses in the last month found a higher proportion of respiratory diagnoses in individuals without psychiatric contact than in those with such contact [12]. Apart from the higher age it is also possible that anxiety symptoms, such as difficulty breathing, feeling of pressure in the chest or palpitations, might have been misread as somatic symptoms. Additionally, it is well documented that depression rates are elevated in patients with cardiovascular diseases [25].

Physical pain emerged as a prevalent symptom in our study, documented in the GP records of approximately one-third of all individuals. This proportion was similar to findings from a previous study, where around 40% of individuals reported physical pain during their last medical consultation prior to suicide [26]. However, it is worth noting that physical pain is also common in the general population, as shown in a population-based study across six European countries where 29% of the respondents reported persistent pain over the previous 12 months [8]. As physical pain is a recognized risk factor for suicide [27], it remains important to assess suicidality in patients with physical pain in spite of it being a common symptom. Previous research has also indicated that depressed men are more inclined to report physical symptoms during GP visits compared to non-depressed counterparts [28], which might additionally point to a need for investigating possible mental health issues in patients reporting physical pain.

### Notations of elevated suicide risk

Almost half of the individuals in this primary care cohort had known psychiatric diagnoses, but elevated suicide risk was noted in only 6% of the total group and just 3% in the group without psychiatric contact. This latter figure can be compared to the findings of Hamdi et al. [13] who assessed GP records during the final 3 months of life. Among persons without prior psychiatric contact in that study, approximately 12% were assessed to be at moderate to high suicide risk, and 4% were considered to be at imminent risk. Another study examining the final pre-suicide care contacts with GPs in the UK reported suicidal communication in 15% of cases in contact with mental health services in the last year, although only 3% were assessed by the GP: s to be at moderate to high suicide risk [29]. This is more consistent with the low figures in our study.

### Referrals to psychiatric specialist services

The referral rate to psychiatric specialist services was notably higher among individuals with a history of prior psychiatric contacts, which aligns with the findings of Hamdi et al. [13]. The study setting comprised all of Sweden, encompassing regions ranging from urban to rural and remote sparsely populated areas. This geographical diversity may influence the referral patterns to psychiatric specialist care; urban areas typically offer easier access to this, as opposed to primary care providers retaining patients longer in the more sparsely populated regions with longer traveling distances to the secondary care facilities.

### Treatment

Nearly half of the individuals in our study were prescribed antidepressant medication. This proportion was considerably higher than observed in the general population in Sweden [30], or compared to the prevalence figures reported in a study of the general population in low-income, middle-income and high-income countries [31]. The finding that nearly half of our cohort were on antidepressants at the time of suicide suggests that a need for mental health treatment was recognized but the treatment was not effective enough to prevent suicide. Although prior research has shown a suicide preventive effect of antidepressants [32, 33], it is important to bear in mind that not all patients respond to treatments with antidepressants. The lack of treatment response in our study may stem from various factors, including inadequate dosage, non-adherence, or only partial response. The average response rate for antidepressants being estimated to approximately 60% [34]. A Swedish study investigating mental health treatments in primary care revealed inadequate medication use, defined as the duration of antidepressant treatment for a common mental disorder less than 6 months, in approximately one third of individuals [35].

According to medication lists, more than half of the suicide decedents in our study were prescribed anxiolytics or hypnotics at the time of death. Considering the mean age of the cohort (58 years) it should be pointed out that anxiolytics tend to be common in older populations, both in Sweden [36] and in other countries [37].

### Implications for care

The finding that many of the individuals in our cohort died by suicide despite the identification and treatment of symptoms of mental ill-health suggests a need for enhanced mental health treatment within primary care. Less than one in five individuals in our study were prescribed psychotherapy or counselling in primary care, which may indicate a greater need for this. Alongside medication and psychotherapy/counselling, other care models such as collaborative care [34], in which primary care personnel collaborate with behavioral health professionals supported by a psychiatric consultant, have demonstrated effectiveness in managing mental health problems within primary care [35]. The system of specialized care managers has been introduced together with primary care nurses in some parts of Sweden [36], but is not yet part of routine care.

### Strengths and limitations

One of the key strengths of this study lies in the utilization of medical records data, which allows comprehensive insights into the nature of primary health care contacts during the final month of life. The personal identification number assigned to all individuals registered in the Swedish health system enabled the identification of all suicide decedents in Sweden, ensuring a high level of coverage. Moreover, the predominantly public-funded and regionally managed health care system in Sweden facilitated access to medical records, ensuring robust coverage [4]. This afforded a rare opportunity to extend findings from register-based research, providing insight into symptomatology, documentation of elevated suicidality as well as treatment planned at the final GP consultation. Limitations are primarily the small sample size and the absence of Inter-Rater Reliability (IRR) testing, which may impact the generalizability and reliability of the findings.

## Conclusion

This study aimed to describe the clinical characteristics of individuals visiting their GP in the 30 days before death by suicide. The findings illustrate that only a small proportion were noted to have an elevated suicide risk at the last GP consultation, despite presenting with psychiatric symptoms and other established clinical and demographic risk factors (e.g. age, gender, somatic symptoms), especially in patients without a known psychiatric history. In other words, psychiatric problems went undetected in a large proportion of individuals and suicidality in an even larger proportion. Another finding was that a large proportion of all individuals presented with somatic symptoms, particularly physical pain. These findings suggest that individuals with somatic symptoms, especially middle-aged and older, require more attention to their psychiatric symptoms including a plan for suicide risk management when applicable.

### Implications for future research

It would be fruitful to explore whether primary health care interventions differ between individuals who do and do not die by suicide, despite similar psychopathology. However, this investigation would require a different study design with matched controls.

### Electronic supplementary material

Below is the link to the electronic supplementary material.

Supplementary Material 1

## Data Availability

Upon reasonable request relevant data are available from the corresponding author.

## Abbreviations

GP: General Practitioner
NPC: No Psychiatric Contact group
PC: Psychiatric contact group

## References

[1] Luoma JB, Martin CE, Pearson JL (2002). Contact with mental health and primary care providers before suicide: a review of the evidence. Am J Psychiatry.

[2] Leavey G, Rosato M, Galway K, Hughes L, Mallon S, Rondon J (2016). Patterns and predictors of help-seeking contacts with health services and general practitioner detection of suicidality prior to suicide: a cohort analysis of suicides occurring over a two-year period. BMC Psychiatry.

[3] Ahmedani BK, Simon GE, Stewart C, Beck A, Waitzfelder BE, Rossom R (2014). Health care contacts in the year before suicide death. J Gen Intern Med.

[4] Bergqvist E, Probert-Lindström S, Fröding E, Palmqvist-Öberg N, Ehnvall A, Sunnqvist C et al. Health care utilisation two years prior to suicide in Sweden: a retrospective explorative study based on medical records. BMC Health Serv Res. 2022;22(1). 10.1186/s12913-022-08044-9 PMC9115926 35581647

[5] John A, DelPozo-Banos M, Gunnell D, Dennis M, Scourfield J, Ford DV (2020). Contacts with primary and secondary healthcare prior to suicide: case-control whole-population-based study using person-level linked routine data in Wales, UK, 2000–2017. Br J Psychiatry: J Mental Sci.

[6] McMahon EM, Greiner BA, Corcoran P, Larkin C, Leitao S, McCarthy J, et al. Psychosocial and psychiatric factors preceding death by suicide: a case–control psychological autopsy study involving multiple data sources. Suicide and Life-Threatening Behavior; 2022. 10.1111/sltb.12900 PMC9796414 35815892

[7] O’Connor RC, Worthman CM, Abanga M, Athanassopoulou N, Boyce N, Chan LF (2023). Gone too soon: priorities for action to prevent premature mortality associated with mental illness and mental distress. Lancet Psychiatry.

[8] Demyttenaere K, Bonnewyn A, Bruffaerts R, Brugha T, De Graaf R, Alonso J (2006). Comorbid painful physical symptoms and depression: prevalence, work loss, and help seeking. J Affect Disord.

[9] Rodi PM, RoŠKar S, MaruŠIČ A, SUICIDE, VICTIMS’ LAST, CONTACT WITH THE PRIMARY CARE PHYSICIAN (2010). REPORT FROM SLOVENIA. Int J Soc Psychiatry.

[10] Draper B, Snowdon J, Wyder M (2008). A pilot study of the suicide victim’s last contact with a health professional. Crisis: J Crisis Intervention Suicide Prev.

[11] Saarinen P, Hintikka, Lehtonen J (1998). Somatic symptoms and risk of suicide / somatic symptoms and risk of suicide. Nord J Psychiatry.

[12] Pan Y-J, Lee M-B, Chiang H-C, Liao S-C (2009). The recognition of diagnosable psychiatric disorders in suicide cases’ last medical contacts. Gen Hosp Psychiatry.

[13] Hamdi E, Price S, Qassem T, Amin Y, Jones D (2008). Suicides not in contact with mental health services: risk indicators and determinants of referral. J Mental Health.

[14] Salander Renberg E, Sunnqvist C, Westrin Å, Waern M, Jokinen J, Runeson B. Suicidnära patienter -kliniska riktlinjer för utredning och vård. Föreningen. SP, editor. Stockholm: Svenska Psykiatriska Föreningen och Gothia Fortbildning AB; 2013 2013.

[15] Swedish National Board of Health and Welfare [Internet]. [cited 16/02/2023].

[16] WHO (1992). WHO International statistical classification of diseases and related health problems: ICD-10.

[17] Brooke HL, Talback M, Hornblad J, Johansson LA, Ludvigsson JF, Druid H (2017). The Swedish cause of death register. Eur J Epidemiol.

[18] Laanani M, Imbaud C, Tuppin P, Poulalhon C, Jollant F, Coste J (2020). Contacts with Health services during the Year prior to suicide death and prevalent conditions a Nationwide Study. J Affect Disord.

[19] Stene-Larsen K, Reneflot A (2019). Contact with primary and mental health care prior to suicide: a systematic review of the literature from 2000 to 2017. Scand J Public Health.

[20] Bakken IJ, Ellingsen CL, Pedersen AG, Leistad L, Kinge JM, Ebbing M, Norwegian (2015). J nor Med Association / Tidsskrift Den Norske Laegeforening.

[21] Ahmedani BK, Westphal J, Autio K, Elsiss F, Peterson EL, Beck A (2019). Variation in patterns of health care before suicide: a population case-control study. Prev Med.

[22] Mann JJ, Michel CA, Auerbach RP (2021). Improving suicide Prevention through evidence-based strategies: a systematic review. Am J Psychiatry.

[23] Doering S, Probert-Lindström S, Ehnvall A, Wiktorsson S, Palmqvist Öberg N, Bergqvist E (2024). Anxiety symptoms preceding suicide: a Swedish nationwide record review. J Affect Disord.

[24] Mellqvist Fässberg M, Cheung G, Canetto SS, Erlangsen A, Lapierre S, Lindner R (2016). A systematic review of physical illness, functional disability, and suicidal behaviour among older adults. Aging Ment Health.

[25] Bradley SM, Rumsfeld JS (2015). Depression and cardiovascular disease. Trends Cardiovasc Med.

[26] Urša Mars B, Lara P, Petra Mesec R, Vita P, Diego De L, NATURE OF THE LAST CONTACT WITH A MEDICAL DOCTOR BEFORE SUICIDE. Innovative Issues and Approaches in Social Sciences. 2018;11(1).

[27] Tang NK, Crane C (2006). Suicidality in chronic pain: a review of the prevalence, risk factors and psychological links. Psychol Med.

[28] Shiels C, Gabbay M, Dowrick C, Hulbert C (2004). Depression in men attending a rural general practice: factors associated with prevalence of depressive symptoms and diagnosis. Br J Psychiatry.

[29] Pearson A, Saini P, Da Cruz D, While D, Swinson N, Williams A (2009). Primary care contact prior to suicide in individuals with mental illness. Br J Gen Pract.

[30] Statistik om läkemedel. 2020 [Internet]. 2020 [cited 20-01-2022].

[31] Kazdin AE, Wu C-S, Hwang I, Puac-Polanco V, Sampson NA, Al-Hamzawi A (2023). Antidepressant use in low- middle- and high-income countries: a World Mental health surveys report. Psychol Med.

[32] Castelpietra G, Gobbato M, Valent F, De Vido C, Balestrieri M, Isacsson G (2017). Antidepressant use in suicides: a case-control study from the Friuli Venezia Giulia Region, Italy, 2005–2014. Eur J Clin Pharmacol.

[33] Isacsson G, Holmgren A, Osby U, Ahlner J (2009). Decrease in suicide among the individuals treated with antidepressants: a controlled study of antidepressants in suicide, Sweden 1995–2005. Acta Psychiatrica Scandinavica.

[34] Mulrow CD, Williams JW, Chiquette E, Aguilar C, Hitchcock-Noel P, Lee S (2000). Efficacy of newer medications for treating depression in primary care patients. Am J Med.

[35] Sandheimer C, Björkelund C, Hensing G, Mehlig K, Hedenrud T. Implementation of a care manager organisation and its association with antidepressant medication patterns: a register-based study of primary care centres in Sweden. BMJ Open. 2021;11(3). 10.1136/bmjopen-2020-044959 PMC7938985 33674375

[36] Swedish National Board of Health and Welfare. Öppna jämförelser 2014, Läkemedelsbehandlingar. 2014.

[37] Landolt S, Rosemann T, Blozik E, Brüngger B, Huber CA. Benzodiazepine and Z-Drug Use in Switzerland: Prevalence, Prescription Patterns and Association with Adverse Healthcare Outcomes. Neuropsychiatric Disease and Treatment. 2021;ume 17:1021-34. 10.2147/NDT.S290104 PMC8052118 33880026

